# 

**Published:** 1889-11-09

**Authors:** 


### NOTICE TO CORRESPONDENTS.

All MS., Letters, Books for Review, and other matters intended for the Editor, should be addressed The Editor, The Lodge, Porchester Square, London, W.

**Notice to Advertisers and Subscribers.**

All Advertisements, Orders for Copies of The Hospital, and Business Communications should be addressed to The Publisher, *not to the Editor*, The Hospital, 139-140, Salisbury-court, Fleet-street, E.C.

**Bound Volumes of "The Hospital."**

Vol. VI., now ready, 4s., post free. Cases for binding, may be had of the Publisher, at 1s. 6d. each.

**To Residents, Secretaries, and Matrons**

The Editor will be glad to have the Regulations and each Report as issued by Asylums, Hospitals, Convalescent and Nursing Institutions, as well as those of other Charities, and an early copy in duplicate of all Appeals and Festival Notices, etc., addressed to *The Editor, The Lodge Porchester-square, London, W.* Any superintendent, secretary, matron, or other chief official who regularly sends such information may be registered, on application to the Publisher, to receive free of cost a cover for binding The Hospital in half-yearly volumes.

### Scraps and Gleanings.

Dr. Heath Strange has been elected president of Hampstead Medical Society.

Dr. Klein is recommended as one of the council of the Royal Society for next year.

The late Viscountess Combermere has left £2,000 to the Earlswood Asylum for Idiots.

Mrs. Bishop has failed so far in her cherished idea of starting a hospital at Nazareth.

The Salvation Army opened a shelter and food depot in the Whitechapel-road last week.

The new Dublin Gymnasium and Physical Training School has Dr. Meldon for vice-president.

Dr. Barry and Staff-Surgeon Preston have been examined by the Royal Commission on Vaccination.

At the Royal Ear Hospital, Frith-street, Soho-square, 744 patients were under treatment during October.

The Local Government Board are to hold an enquiry concerning the alleged scandals about the Islington Workhouse.

Surgeon Simeon Le Quesne has been presented with the Victoria Cross for conspicuous bravery in Upper Burmah.

Hospital Saturday at Swindon resulted in a cheque for £72 being sent to Swindon Victoria Hospital. This is good.

The New York Society of Medical Jurisprudence has expressed its sympathy with Drs. Irwin, Ferguson, and Hance.

Mrs. Liddell has subscribed £300 to Hull Royal Infirmary, and £100 to the Victoria Hospital for Poor Sick Children, Hull.

Mr. John Sherburn, M.R.C.S., on the invitation of the Hull Town Council, has consented to hold the mayoralty for another year.

Mrs. Ellen M. Gifford, of New Haven, who died Sept. 7th, has left the whole of her large fortune to various charities in the United States.

New York Asylum for Lying-in Women treated 136 in-patients last year without one death. The sixty-sixth annual report is just published.

Mr. Robert W. Reid, M.D., has been appointed Professor of Anatomy in the University of Aberdeen, in the room of Professor Struthers, resigned.

Mr. E. Maynouth Reid has been elected Professor of Physiology at Dundee. Dr. Reid is Assistant Lecturer on Physiology at St. Mary's Hospital.

The German "Frauverein" for the care of the sick in the colonies will hold a bazaar shortly, funds being needed by their East African hospitals.

The Harveian Lectures, on the "Surgery of the Kidney," will be delivered by Mr. Knowsley Thornton, F.R.C.S.Eng., on Nov. 21st and 28th and Dec. 5th.

The School Board is accused of causing brain over-pressure, and now the telephone is accused of causing aural over-pressure and consequent diseases of the ear.

Dr. Kebbell, medical officer of health for Hove, in his report for the quarter ending September 30, states that the number of deaths was 59, being at the rate 8·7 per 1,000.

In spite of Lord Meath's absence from England, the Metropolitan Public Garden's Association is working as vigorously as ever. The organisation must be excellent.

Medical degrees in the Czar's dominions will henceforth only be given to women who have studied in Russia. No woman under 40 will be allowed to practice as a doctor.

A poor woman, named Spicer, the wife of a labourer at Great Hormead, Herts, lately gave birth to four children. The woman and three of the children died the same evening.

Prof. Milo de Meyer is giving a course of experimental lectures on Practical Magnetism and Hypnotism to the members of the Magnetic and Hypnotic Society of Great Britain.

New York Board of Health engages forty doctors every summer to visit from house to house in tenement districts. Last summer 3,826 houses, containing 25,746 families, were visited.

An International Exhibition of Electrical Engineering will be held in Edinburgh next year. There will be a section for electrical medical, and surgical apparatus, also a section for hygiene.

Lady Brooke, the founder of the Essex Needlework Guild, recently appealed in the local papers for assistance, and last week upwards of 4,000 garments have been received at the Town Hall, Dunmow.

Mr. Evan Spicer, chairman of the South London Polytechnic Institutes, has received a promise of £1,000 from Mr. Frederick Nettlefold, of Streatham, towards the amount required for the Battersea Polytechnic.

In King's College Hospital, the committee of management have just been able to reopen a ward of 13 beds, being one of two which was closed in 1885 for lack of funds. The ward will be devoted to the relief of women.

The grant of the four extra pensions of £100 per annum each to the senior officers sanctioned in clause 91 Indian Army Circulars, 1886, will be discontinued in the case of all officers appointed in future to the Indian medical establishment.

The Queen of Greece, with the Empress Frederick and her daughters, and the Princess of Wales and her daughters, visited several charitable institutions in Athens. Among the places inspected were the Establishment for Poor Women and the Queen's Hospital.

At St. Thomas's Hospital on the 26th ult., Mr. Sidney Plowman, who is leaving the hospital, having accepted the appointment of Professor of Pharmacology, etc., to the University of Melbourne, was presented with the new edition of the "Encyclopædia Britannica" in an open case.

The report of the Hastings medical officer of health, for the quarter ended September 30th last, states that the death rate for the quarter, calculated upon an estimated population of 57,176 was 12·55 per 1,000, being 0·92 per 1,000 below the average for this quarter for the last eight years.

